# Research Progress of Natural Products and Their Derivatives in Marine Antifouling

**DOI:** 10.3390/ma16186190

**Published:** 2023-09-13

**Authors:** Wenwen Zhao, Zhiqiang Wu, Yanming Liu, Pan Dai, Guojuan Hai, Feng Liu, Yu Shang, Zhongyue Cao, Wufang Yang

**Affiliations:** 1 Xi’an Key Laboratory of High Performance Oil and Gas Field Materials, College of Materials Science and Engineering, Xi’an Shiyou University, Xi’an 710065, China; 2 Center of Advanced Lubrication and Seal Materials, School of Materials Science and Engineering, Northwestern Polytechnical University, Xi’an 710072, China; 3 State Key Laboratory of Solid Lubrication, Lanzhou Institute of Chemical Physics, Chinese Academy of Sciences, Lanzhou 730000, China

**Keywords:** natural products, biofouling, marine antifouling

## Abstract

With the increasing awareness of environmental protection, it is necessary to develop natural product extracts as antifouling (AF) agents for alternatives to toxic biocides or metal-based AF paints to control biofouling. This paper briefly summarizes the latest developments in the natural product extracts and their derivatives or analogues from marine microorganisms to terrestrial plants as AF agents in the last five years. Moreover, this paper discusses the structures–activity relationship of these AF compounds and expands their AF mechanisms. Inspired by the molecular structure of natural products, some derivatives or analogues of natural product extracts and some novel strategies for improving the AF activity of protective coatings have been proposed as guidance for the development of a new generation of environmentally friendly AF agents.

## 1. Introduction

Marine biofouling is an undesirable phenomenon that often involves the adhesion and colonization of microfouling and macrofouling on man-made substrates [1,2]. Fouling organisms are mainly including microorganisms (such as bacteria, fungi, diatoms, etc.) and macroorganisms (such as mussels, sponges, hydroid, seaweeds, etc.). The accumulation of organisms can significantly increase the fuel consumption and the maintenance costs, pose a safety hazard and induce the invasion of alien species [3,4,5]. At present, among various AF methods, coatings AF layer on the substrate surface is undoubtedly the most effective and easy way. Over the past decade, various AF agents have been developed, such as copper and copper compounds, zinc, Irgarol 1051 and Sea-Nine 211, since the use of triphenyltin (TBT) system AF coatings have been banned in the worldwide due to their environmental toxicity [6,7,8]. However, the release of metals from copper- and zinc-based AF coatings are also deleterious to marine organisms which are widely used in the industry [9,10,11,12]. Therefore, based on the adverse effects of excessive release of metal ions in the widely used AF coatings on the development of marine ecosystems, it is of great significance to develop environmentally friendly coatings with nontoxic AF agents.

Natural product extracts are potential alternatives to replace toxic AF agents. Marine organisms, such as corals, sharks and marine plants, can prevent their body surfaces from AF substances without causing serious environmental problems [13,14]. On this basis, scientific and technological workers have conducted a series of related studies on marine microorganisms [15], marine invertebrates [16] and aquatic plants [17] as well as terrestrial plants such as pepper [18,19], *Stellera chamaejasme* [20] and coffee [21]. Various natural agents with good AF properties have been extracted from these organisms. Meanwhile, the natural product extracts from different organisms have different chemical compositions and molecular structures, exhibiting different AF properties for different application scenarios. This paper will provide an overview of the latest developments in AF compounds isolated from marine organisms and terrestrial plants in the last five years and expand their AF mechanisms. The aim of this work is to briefly summarize the structures–activity relationship of these AF compounds and their derivatives or analogues, further providing guidance for the development of a new generation of environmentally friendly AF agents.

## 2. Compounds from Marine Organisms

### 2.1. Compounds from Algae

At present, relevant studies on the natural product extracts from algae mainly focus on algae, such as *Asparagine armata*, *Sargassum*, *Posidonia oceanica* and *Laurencia venusta*. Some novel compounds including terpenoids, diterpenoids, phenolic compounds and their derivatives as well as some green biosynthesis methods have been continuously reported as substitutes or strategies for the next generation of AF agents. Some compounds extracted from algae are shown in Table 1.

**Figure 1:**
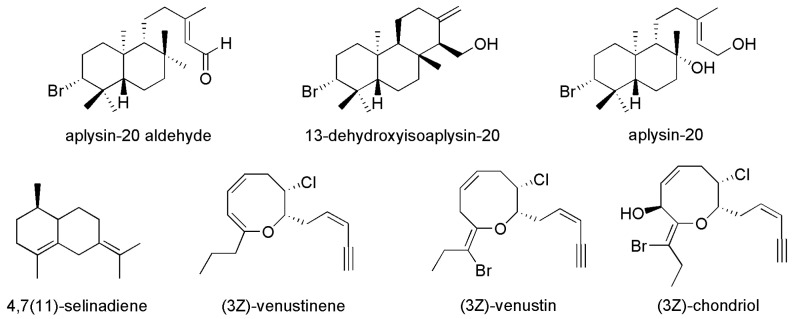
Structures of Aplysin-20 aldehyde (1), 13-dehydroxyisoaplysin-20 (2) and its congeners (3–8) collected from the marine red algal genus *Laurencia venusta*. Reprinted with permission from [22].

Takashi Kamada’s group reported two novel brominated diterpenoids, aplysin-20 aldehyde, 13-dehydroxyisoaplysin-20 and its congeners collected from the marine red algal genus *Laurencia venusta* (Figure 1). The Aplysin-20 aldehyde, 13-dehydroxyisoaplysin-20 and aplysin-20 exerted a strong inhibitory effect on the mussel *Mytilus galloprovincialis* [22]. *Asparagopsis armata* and *Sargassum muticum* are two invasive species which occur at several coastlines all over the world [23]. Some crude extracts of these two seaweeds were collected, and all these compounds inhibited growth of bacteria and microalgae, also reducing the formation of bacterial biofilms [24]. Studying the AF active substances of these invasive species can not only use invasive species as a source of AF agents but also reduce the negative effects of biological invasion. *Posidonia oceanica* is one of the most representative organisms of the Mediterranean Sea and contains hundreds of bioactive compounds. Its leaf extract exhibited the highest AF activity against growth of *Phaeodactylum tricornutum* with an EC_50_ (the half maximal effective concentration) values of 51.62 mg/L and inhibited the *Aliivibrio fischeri* bioluminescence at a concentration of 2.813 mg/L [17]. The antibiofouling activities of the hydroalcoholic extract of *Posidonia oceanica* were attributed to the synergistic action of its phenolic compounds, making it viable as an additive to fouling release formulations. The extract contained phenolic hydroxyl groups, which can bind proteins, and then denature or precipitate proteins or inactivate enzyme systems, thereby exhibiting antifouling activity [25]. Hence, *Posidonia oceanica* extract provided suitable levels of AF activity against bacteria (*Aliivibrio fscheri*), diatoms (*Phaeodactylum tricornutum*) and serpulid polychaetes (*Ficopomatus enigmaticus*), and significantly reduced adhesion of *Navicula salinicola* cells and facilitated their release in low-intensity water flows.

**Figure 2:**
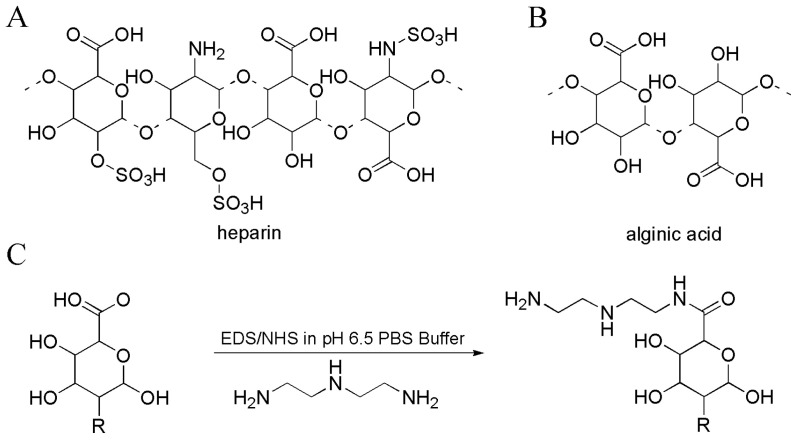
Molecular structures of the main building blocks of (**A**) heparin and (**B**) alginic acid. (**C**) The carboxylic acid functions of the PSs were modified with DETA using an EDC/NHS (N-(3-dimethylaminopropyl)-N′-ethylcarbodiimide, N-hydroxysuccinimide) coupling at pH 6.5 in PBS buffer. Reprinted with permission from [26].

**Figure 3:**
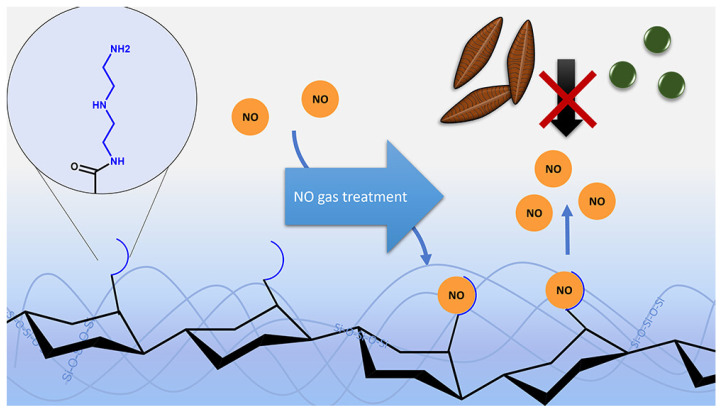
AF mechanism schematic illustration of the hybrid material coatings contained amino groups. Reprinted with permission from [26].

Axel Rosenhahn et al. prepared polysaccharide-based hybrid material coatings (hybrid polymers from polysaccharides (PSs) and silanes) by combining diethylenetriamine(DETA)-modified heparin and alginates, as well as unmodified polysaccharides (Figure 2) [25]. The hybrid material coatings contained a large amount of amino groups which can absorb nitrogen oxide (NO) at elevated pressures and showed NO release rates of 17–30 pmol·cm^−2^·s^−1^ in aqueous environment (Figure 3). DETA is polyamine precursor molecule which can form N-nitrosamines and N-diazeniumdiolates (NONOates) with NO. NO can be released from hybrid material coatings due to the protonation of NONOates into water. Hybrid materials with NO release exhibited better AF properties against the marine bacterium *Cobetia marina* and the diatom *Navicula perminuta* than hybrid materials coatings without NO release. NO-releasing coatings exhibited AF activity by NO-induced dispersal of biofilms. Due to their low toxicity and good biocompatibility, NO-releasing coatings may be interesting alternatives to conventional, biocidal technologies.

As paints additives with different weight on different substrates, nanoparticle compounds were prepared using chitosan, *Ulva fasciata*, and *Avicennia marina* leaves extracts by a green biosynthesis method [27]. These biosynthesized nanoparticles (*Avicennia marina*) showed the highest AF activity at different periods, and still maintained good AF performance after being immersed in seawater for 7 months. The superior AF performance of *Avicennia marina* leaves extract can be owning to its constituents of polyphenols, ammonium compounds and high concentrations of alcohols, besides the presence of both aromatic and aliphatic amide and amide derivatives.

### 2.2. Compounds from Marine Invertebrates

Marine invertebrate account for the majority of marine animals, and they have a wide range of species, including nearly 20 animal phyla. Here we mainly introduce the recent research progress in natural extracts from sponges, corals and other coelenterates. Table 2 shows some compounds extracted from marine invertebrates.

**Figure 4:**
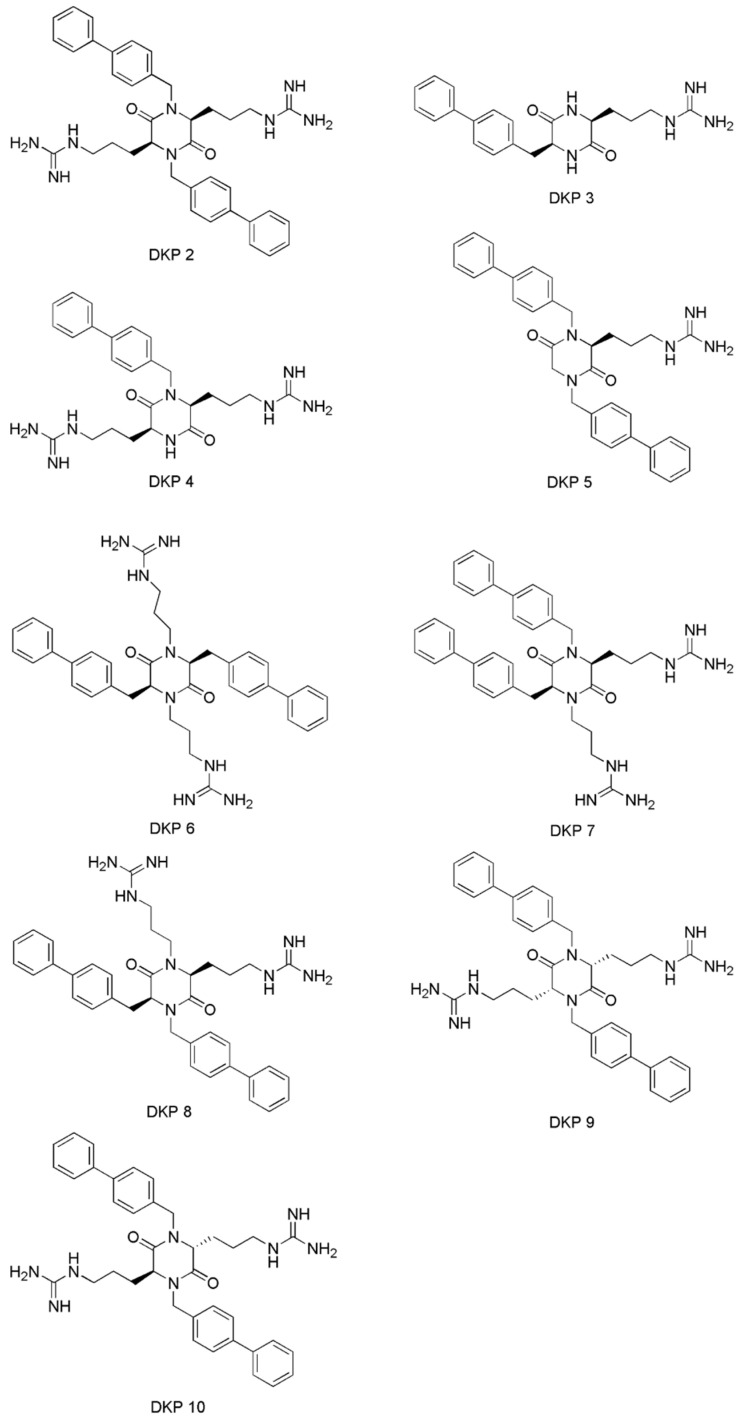
The molecular structures of the synthesized cationic amphiphilic 2,5-DKP regioisomers. Reprinted with permission from [37].

**Figure 5:**
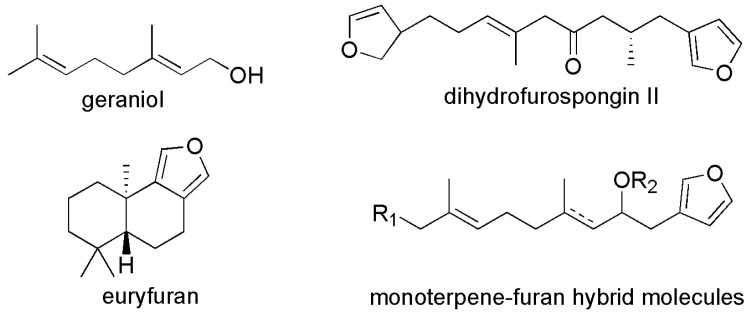
The molecular structures of geraniol, dihydrofurospongin II, euryfuran and monoterpene-furan hybrid molecules [31].

Two main compounds isolated from the sponge *Dendrilla antarctica* were 9,11-dihydrogracillinone A and 9,11-dihydrogracilin A, and these two AF agents can effectively inhibit the settlement of a variety of colonizing organisms [28]. The main compound isolated from the Patagonian sponge *Siphonochalina fortis* was peracetylated cholic acid, which exerted AF activity against the mussel *Mytilus edulis platensis* and had low toxicity [29]. Barettin containing 2,5-diketopiperazine (DKP) ring was isolated from marine sponge *Geodia barretti* and possessed the ability to bind to a range of receptors due to its privileged structures [30]. Grant et al. incorporated the pharmacophore derived from amphiphilic micropeptides into DKP and synthesized a library of amphiphilic 2,5-DKPs, which exhibited broad-spectrum activity [36]. Subsequently, the group developed a synthetic method to prepare tetrasubsituted 2,5-DKP regioisomers and synthesized novel nine compounds (Figure 4), which showed good AF activities against *Ciona savignyi*, *Mytilus galloprovincialis*, *Spirobranchus cariniferus* and *Undaria pinnatifida* except DKPs 4 [37]. DKP 5, with two hydrophobicity regions and one positive charge region, displayed the best AF performance. Hydrophobicity region and positive charge region were attributed to biphenyl groups and arginine sidechain, respectively. The calculated value of cLog*D*_8.1_ (liposolubility) of DKP 5 was positive, which indicated that DKP 5 can effectively act on the cell membrane of fouling organisms. DKP 4 possessed two positive charge regions and one hydrophobicity region, and the calculated value of cLog*D*_8.1_ was negative. It indicated that DKP 4 was more hydrophilic than DKP5, and therefore, it was inactive against macrofouling organisms. It was demonstrated that the broad-spectrum AF of this class of versatile compounds was dictated by the balance between hydrophobicity and cationic charge. Takamura, H et al. synthesized nine monoterpene–furan hybrid molecules by inspiring the structure of geraniol (a naturally occurring monoterpene) and furan isolated from *Mediterranean sponges* (Figure 5) [31]. These monoterpene–furan hybrids inhibited the settlement of the cypris larvae of the barnacle *Balanus* (*Amphibalanus*) *amphitrite* with EC_50_ values of 1.65–4.70 μg·mL^−1^.

**Figure 6:**
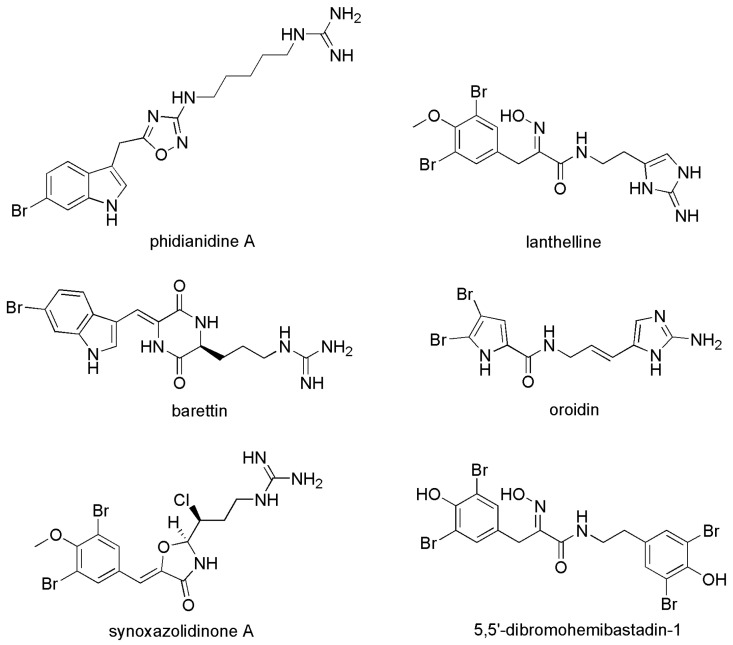
The molecular structures of phidianidine A and a selection of analogues with inhibitory concentration (IC_50_) included [38].

Phidianidine A extracted from the aeolid opisthobranch mollusk *Phidiana militaris* was structurally and chemically significantly analogous to ianthelline, barettin and the synoxazolidinones (Figure 6) [32]. These structural features often linked to a high AF activity. Based on this point, Johan Svenson’s group synthesized a series of analogues and investigated inhibitory activities against the settlement and metamorphosis of *Amphibalanus improvisus* cyprids [38]. The experimental results showed that phidianidine A was nontoxic and exhibited strong AF activity against barnacle cyprid metamorphosis. The bioactivity of synthetic analogues is closely related to the size of the compound and its basicity. The study also illustrated that active analogues can be prepared in the absence of the natural constrained 1,2,4-oxadiazole ring, and showed settlement inhibition of *Amphibalanus improvisus* cyprids with IC50 (the half maximal inhibitory concentration) values of 0.7 μg/mL.

**Figure 7:**
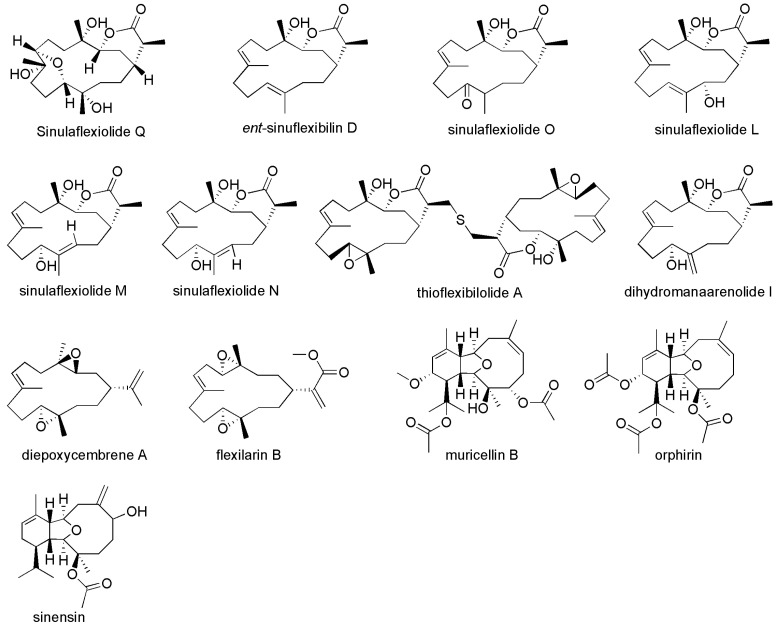
The molecular structures of compounds isolated from *Sinularia flexibilis* and *Muricella* sp. [35].

Corals are a significant source of natural products. Guoqiang Li’s group isolated nine new cembrane diterpenes and three known analogues from the South China Sea soft coral *Sarcophyton glaucum* [33]. It was demonstrated that Sarcoglaucin B, sarcoglaucin E, trochelioid, 7α-hydroxy-Δ^8(19)^-deepoxysarcophine and (−)-sartrochine showed an antilarval settlement activity against *Balanus amphitrite* with adhesive rates of 6.52%, 4.60%, 8.19%, 14.14% and 7.78% at 25 ppm, respectively. Song and coworkers isolated about 200 strains from coral (*Pocillopora damicornis*) to investigate their ability to inhibit quorum sensing [34]. The result showed that 15% of the strains exhibited QSI activity (quorum sensing inhibitor). H12-Vibrio alginolyticus was chose to investigate the mechanism of QSI activity further. It was found that the extract of H12-Vibrio alginolyticus, rhodamine isothiocyanate and its analogue could inhibit the biofilm formation of *Pseudomonas aeruginosa* PAO1 by disturbing quorum sensing regulatory genes and virulence-related genes. Feng‘s group isolated a new compound (1) from the soft coral *Sinularia flexibilis*, and tested the compound, another nine cembranoids (2–10) from *S. flexibilis* and three eunicellin-type diterpenoids (11–13) from the gorgonian *Muricella* sp. for AF activity against larval settlement of the bryozoan *Bugula neritina* (Figure 7) [35]. Experimental results showed that ent-sinuflexibilin D, sinulaflexiolide O, sinulaflexiolide L diepoxycembrene A, orphirin and sinensin significantly inhibited the settlement of *Bugula neritina* larvae, with EC_50_ values of 18.2, 99.7, 67.9, 35.6, 33.9 and 49.3 μM, respectively. By comparing the AF performance of the above compounds, it was found that the difference in AF performance was caused by double bonds and hydroxyl groups. Comparing *ent*-sinuflexibilin D and sinulaflexiolide L, the results showed that the hydroxyl group at the C-13 position in sinulaflexiolide L reduced its AF activity against *Bugula neritina*.

### 2.3. Bacterial and Fungal

**Figure 8:**
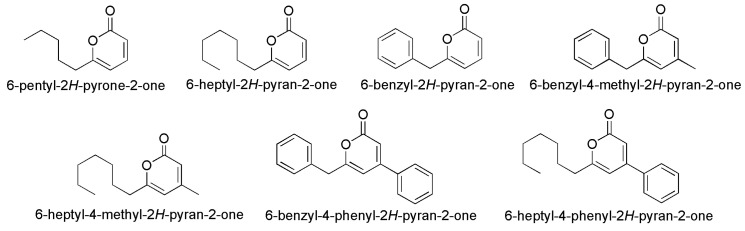
The molecular structure of natural product 6-pentyl-2*H*-pyrone-2-one and synthesized pyrone derivatives [39].

*Trichoderma atroviride* isolated from *Niphates* sp. can inhibit the settlement of barnacle cyprids, because it contains a pyrone-type compound degrading cell walls. Chih-Chuang Liaw’s group isolated one pyrone-type compound (1) (6-pentyl-2H-pyrone-2-one) from the marine-derived *fungi Trichoderma atroviride* and *T. reesei* and demonstrated that this compound had significant inhibitory activities toward barnacle cyprid settlement. Based on this point, they synthesized a series of pyrone analogues and examined the AF properties against barnacle cyprids (Figure 8) [39]. Pyrone dericatives, 6-benzyl-4-phenyl-2H-pyran-2-one and 6-heptyl-4-phenyl-2H-pyran-2-one, exhibited broad spectrum AF properties in barnacle cyprid settlement assays, biofilm formation and antimicrobial assays of marine bacteria by disturbing microbial cell-to-cell communication.

**Figure 9:**
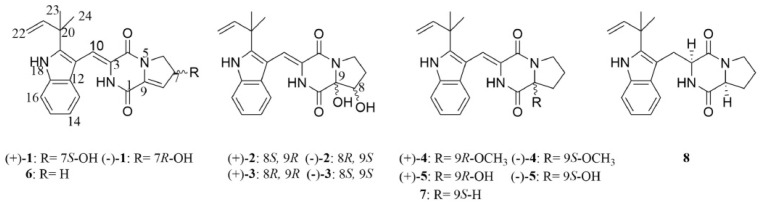
The molecular structures of DKP alkaloids [40].

*Aspergillus versicolor* is also one of the sources of natural product AF agents. Two new pairs of DKP alkaloids ((±)-brevianamide Z[(±)-1] and (±)-brevianamide Z1[(±)-2]), and nine known congeners [(±)-3, (±)-4, (±)-5, 6, 7, and 8] were obtained from the fungus *Aspergillus versicolor* HBU-7 (Figure 9) [40]. In these compounds, (+)-brevianamide V (compound 7) showed significant cytotoxic activity against the HGC-27 cell line, with an IC_50_ value of 4.54 μM. Xiuqin Bai’s group prepared a physical–biological synergistic AF coating by using dopamine as a coupling agent for grafting nisin onto the glass surface, which showed good AF activity against the settlement of *Phaeodactylum tricornutum* and *Bacillus* sp. [41]. H. A. Ibrahim’s group isolated chitosan from marine-derived *Penicillum spinulosum* MH2 cell wall and detected its antimicrobial and AF properties [42]. The extracted chitosan exhibited considerable AF activity against fouling bacteria and showed good antimicrobial activity against *S*. *aureus*, *B*. *subtilis*, *F*. *solani*, *R*. *solani*, *P*. *nutatum*, and *C*. *albicans*. An environmentally friendly AF coating was prepared with the polymer and butenolide derived from the metabolites of marine bacteria [43]. The adhesion strength of the polymer was about 2.0 MPa. The coating displayed good inhibition of the adhesion of marine bacteria *Pseudomonas* sp.

## 3. Compounds from Terrestrial Plants

The extracts from terrestrial plants with AF properties mainly include isothiocyanate compounds, natural organic acids, tannic acid, polylactic acid, capsaicin and their derivatives. The studies on these natural extracts and their derivatives are currently hot topics in marine AF field. Table 3 shows some compounds extracted from terrestrial plants.

**Figure 10:**
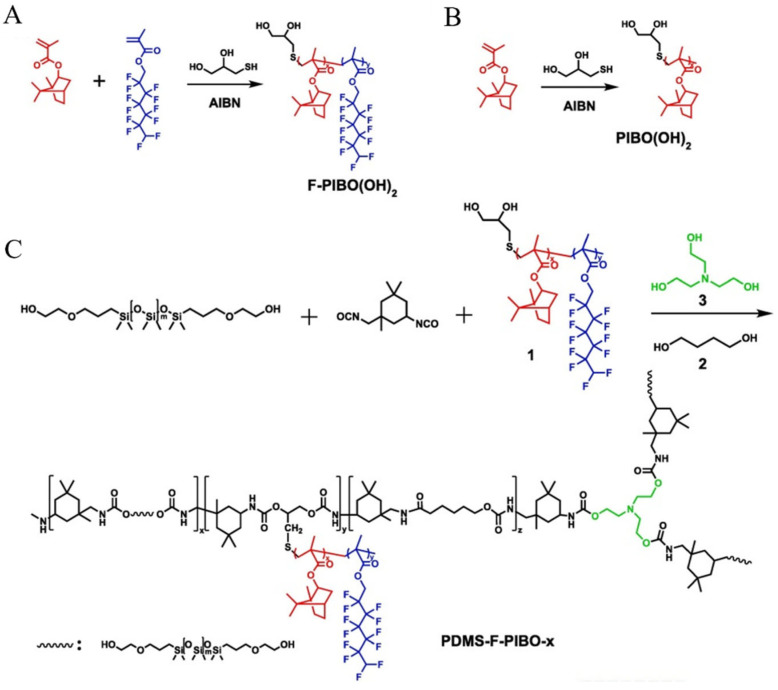
Synthesis process of F-PIBO(OH)_2_ (**A**), PIBO(OH)_2_ (**B**) and PDMS-F-PIBO-x (**C**). Reprinted with permission from [45].

Isothiocyanate compounds isolated from some vegetables have been considered as promising low-toxic AF agents. Yoshikazu Kitano’s group synthesized fifteen β-citronellol-derived isothiocyanate compounds and analyzed their structure–activity relationships [46]. All the synthesized isothiocyanate compounds exhibited effective AF activities, with high therapeutic ratios (LC_50_/EC_50_ > 30). In particular, isothiocyanate compounds with amide or isocyano group revealed a good level of activity against cypris larvae of *Amphibalanus Amphitrite*, with EC_50_ values ranging from 0.1 to 0.32 μg·mL^−1^. Guillermo Blustein’s group isolated five alkaloids from ‘Guatambú’ Trees of the Atlantic rainforest [44]. Among these compounds, N-methyltetrahydroellipticine (isolated from *Aspidosperma austral*) displayed potent AF activity against the settlement of *Mytilus edulis platensis* (*M. edulis platensis*), with EC_50_ of 1.78 nmol/cm^2^. The EC_50_ of furoquinoline alkaloids kokusaginine and flindersiamine (isolated from *Balfourodendron riedelianum*) were 3.86 and 5.56 nmol/cm^2^, respectively. Silicon self-cleaning AF (PDMS-F-PIBO-x) coatings with different mass of isophorone diisocyanate were synthesized by introducing isobornyl as AF group and dodecfluroheptyl into the silicon side chains (Figure 10) [47]. The adhesion strength between coatings and steel substrate was >1 MPa, which meets the needs of daily use. The results showed that PDMA-F-PIBO-23 coating displayed the highest AF activities against attachment of *Pseudomonas* sp. with the detachment efficiency of 95.6%, and inhibited the formation of biofilm.

Based on menthol and natural organic acids, Susana P. Gaudêncio and Ana Rita Cruz Duarte’s group designed hydrophobic deep eutectic systems (HDES) and identified the most appropriate molar ratio and intermolecular interactions for HDES formations by encompassing the physicochemical characterization of different formulations [48]. When the molar ratio of menthol to oleic acid was 1:1, the system possessed the best AF performance against *Mytilus edulis* mussels and *Patella vulgata* limpets. These results proved the potential of the HDES to be sustainable and efficiently used in marine fouling control technologies.

**Figure 11:**
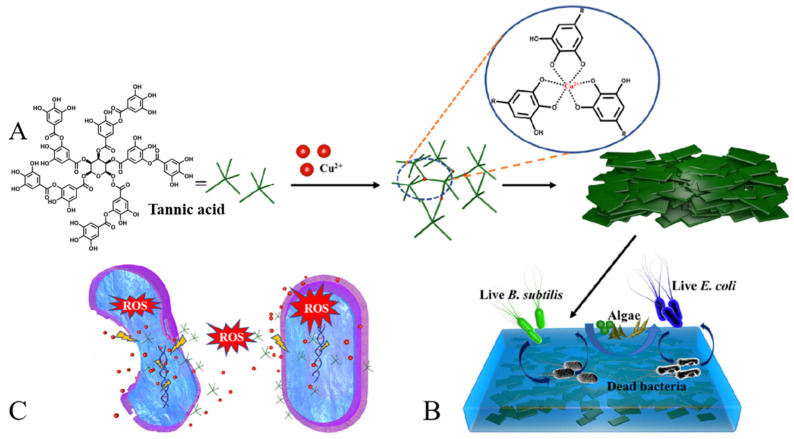
(**A**) the preparation of CuTA nanosheets and (**B**) AF and (**C**) synergetic antibacterial effects of CuTA nanosheet-based coatings against bacteria and algae (ROS refers reactive oxygen species, such as OH^−^, O^2−^ and H_2_O_2_). Reprinted with permission from [56].

Tannic acid (TA) as a natural organic compound widely present in plants has various biological activities, such as antioxidant, anti-inflammatory and antibacterial effects, because it has abundant phenolic hydroxyl groups, which can interact with various small molecules including alkaloids, polysaccharides, metallic ions and proteins [49,50,51]. It can be extracted from the black wattle tree, chestnut wood, oak bark and pines [52,53]. TA is phenolic compounds, and its AF mechanism is similar to the hydroalcoholic extract of Posidonia oceanica mentioned above. Recently, En-Tang Kang’s group reported a bifunctional TA-scaffolded polymer brush coating with a simple “one-step” immersion coating process for AF and antimicrobial applications. Subsequently, this group integrated the pH-responsive strategy, self-defensive mechanism and “one-step” anchoring process to develop an environmental-friendly, stable, and sustainable “smart” coatings [54]. This coatings consist of TA modified with pH-sensitive poly(2-diisopropylaminoethyl methacrylate)- b-poly(2-methacryloyloxyethyl phosphorylcholine) (PDPA-b-PMPC) and cationic polylysine (PLYS) chains (PLYS-TA-PDPA-b-PMPC) anchored on stainless steel surface. The functional surface displayed broad-spectrum AF activities against bacterial, protein and microalgal (*Amphora coffeaeformis*). Kai Zhang and Liqun Xu’s group synthesized natural polyphenol tannic acid (TA)-capped silver nanoparticles (TA–Ag NPs) by an environmentally friendly and sustainable one-step redox reaction under UV irradiation [55]. TA-Ag NPs were deposited on polydimethylsiloxane (PDMA) and other substrate surfaces and exhibited good bacterial-killing performance due to the Ag particles. In addition to modifying plane surfaces, TA can also modify linear and granular materials. Li prepared the copper tannic acid (CuTA) nanosheets and the CuTA/acrylic resin hybrid AF coating (Figure 11) [56]. CuTA powder exhibited strong antibacterial activity against *Bacillus subtilis* and *Escherichia coli*, with a killing rate close to 100% after 24 h (the concentration of CuTA power were 0.5 mg/mL and 2 mg/mL). The CuTA/acrylic resin coating exhibited excellent antimicrobial adhesion performance against *tricornutum*, *Chlorella*, and *Navicula* with 5 wt% CuTA powder. TA-functionalized carbon nanotubes (CNT@TA) embedded with silver nanoparticles can effectively inhibit the settlement of *E. coli* and bovine serum albumin [57]. Feng Zhou’s group prepared a Janus hydrogel wet adhesive through combining poly(vinyl alcohol)/glycerol–tannic acid/Cu^2+^ (PVA/Gly-TA/Cu^2+^) hydrogel with the underwater adhesive poly(dopamine methacrylamide-co-methoxyethyl acrylate) (P(DMA-co-MEA)) via the coordination effect between Cu^2+^ and catechol [58]. This Cu-rich Janus hydrogel showed a significant inhibitory effect on the growth of algae. In addition, the presence of Cu^2+^ improved the mechanical properties of the Janus hydrogel, reaching the adhesion strength of 14 kPa in seawater.

**Figure 12:**
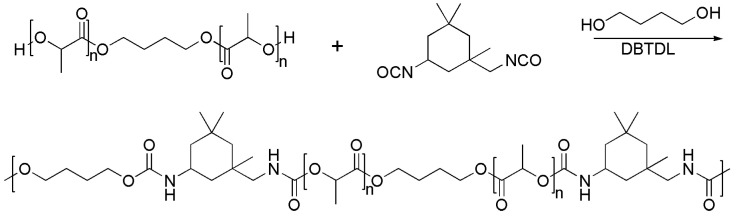
Synthesis of PLA-based polyurethane, where 50% of its polymer chain consists of soft segments (PLA-PU50). Reprinted with permission from [60].

**Figure 13:**
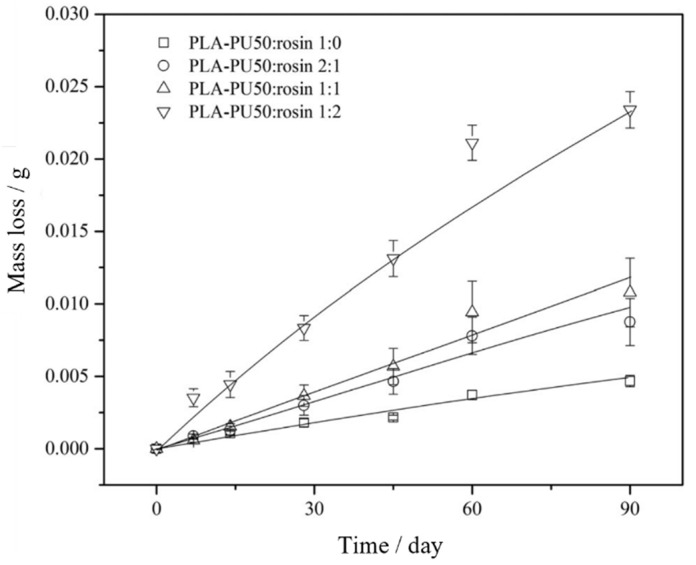
Time-dependent mass loss of PLA-PU50/rosin coatings in ASW over a period of 90 days. Polymer-to-rosin ratios were 1:0, 2:1, 1:1 and 1:2. Reprinted with permission from [60].

Polylactic acid (PLA), which can be extracted from corn, starch and sugarcane, is one of the most widely used biobased and biodegradable polyesters [59]. In the marine environment, PLA can be degraded to LA and then to carbon dioxide and water as final products through the hydrolysis of the ester linkages and enzymatic reactions with microorganisms. Thus, the surface self-renewal ability of the PLA-based coatings and the steady controlled release of AF agents are very crucial for extending the service life of AF coatings. Qian’s group developed a nontoxic biodegradable AF coating (PLA-PU50) by adding a nontoxic AF compound (butenolide) to a PLA-based polyurethane (Figure 12) and investigated the release rate of butanolide, because the biodegradation of PLA was helpful to release butanolide from the AF coating [60]. The result showed that the release rate of butanolide was proportional to the concentration of butanolide in the coating, and incorporating rosin into the coatings increase the self-renewal rate of the polymer further facilitating the long-term release of butenolide from the coating (Figure 13). Pei-Yuan Qian and Chunfeng Ma’s group synthesized biosourced PLA-based polyurethane with hydrolyzable triisopropylsilyl acrylate (TSA) side groups through thiol–ene reaction and polyaddition [59]. This polymer coating can effectively inhibit the adhesion of marine bacteria *Pseudomonas* sp. and had excellent AF ability for more than 3 months as well as a controlled degradation rate tuned by varying its soft segment and TSA content. The ethanol extract of *Stellera chamaejasme* (SC) encapsulated in polydopamine (PDA) microcapsules realized the controlled release in weak alkaline environment [20]. The AF coatings with SC@PDA microcapsules showed good AF activity against *Porphyridium* sp. and *Navicula* sp., with adhesive rates of 15.85% and 20.5%, respectively.

**Figure 14:**
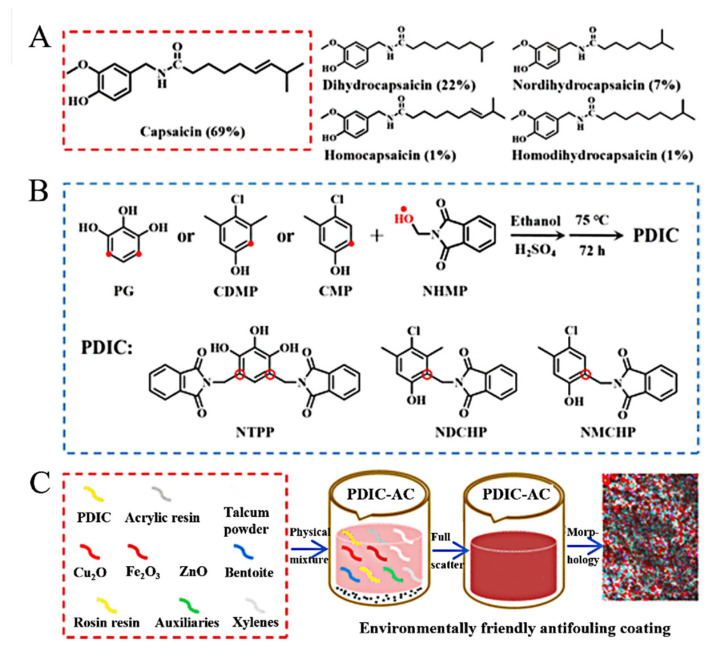
Structure of capsaicinoids (**A**) and preparation of PDIC (**B**) and PDIC-AC (**C**). Reprinted with permission from [18].

**Figure 15:**
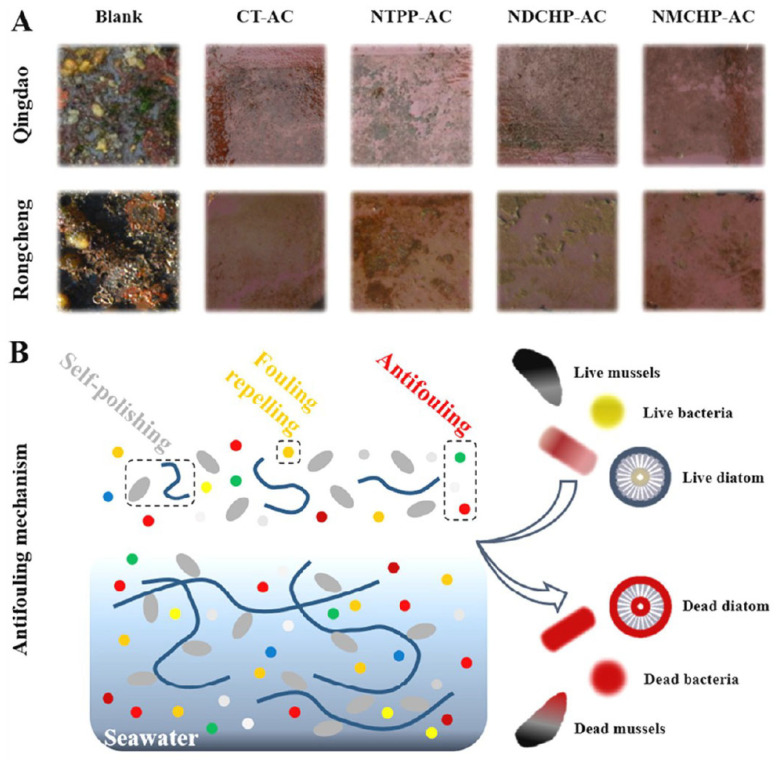
(**A**) AF performance of the PDIC-AC at Qingdao and Rongcheng for 9 months, (**B**) AF mechanism of PDIC-AC. Reprinted with permission from [18].

Because of nontoxicity, environmental friendliness and good AF performance, capsaicin and its derivatives have been used as potential alternatives of toxic AF agents. Inspired by the structure of capsaicin, Liangmin Yu’s group designed eight low-toxicity capsaicin derivatives, which possessed broad-spectrum AF activities against bacterial and algae [19]. The potent AF performance of CAP derivatives was attributed to the main active groups including phenolic hydroxyl group, benzene ring, amide group and chlorine atom. Three new green and high-efficiency AF coatings containing phthalimide derivatives inspired by capsaicin (PDIC-AC) were prepared using a collaborative strategy that incorporates self-polishing, fouling repelling and AF properties (Figure 14) [18]. Due to the changes of roughness, surface free energy and mass loss, the zinc acrylate resin of the PDIC-AC exerted excellent self-polishing properties. Zinc ions in the coatings reacted with sodium ions to form hydrophilic groups in seawater. When the hydrophilic groups accumulated to a certain extent, they were stripped from the main chain to achieve ‘self-polishing’ of the coatings (Figure 15). Both PDIC and PDIC-AC showed excellent inhibitory effect of *E. coli* and *S. aureus*, with the inhibition rates >83%. The test results in the sea areas for 9 months showed that there was no film bubble formation and shedding on the surface of each coating, indicating that the coatings had good stability. Xinglin Guo’s group prepared double-network (DN) hydrogels combining a derivative of capsaicin N-(4-hydroxy-3-methoxybenzyl) acrylamide (HMBA) and polyvinyl alcohol (PVA) [61]. The DN hydrogel can exhibit excellent mechanical strength, low swelling rate, strong oleophobic and excellent AF effect by adjusting the content of HMBA. Shougang Chen’s group designed a series of capsaicin-based pH-triggered polyethylene glycol/capsaicin@chitosan (PEG/CAP@CS), polyvinyl alcohol (PVA)/CAP@CS and alginate (ALG)/CAP@CS multilayer films [62]. All these three types of films exhibit extraordinary pH responsive properties and realize the controlled release of the CAP at a low level in alkaline solutions and at a fast level in acid solutions. The ALG/CAP@CS film showed the best-controlled release performance and long-term antibacterial properties in marine environment.

**Figure 16:**
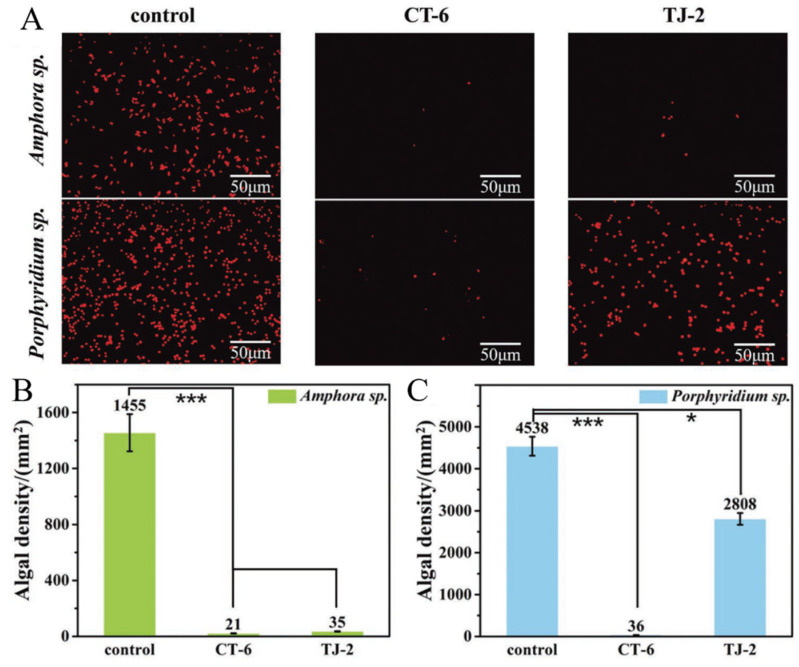
(**A**) The fluorescence pictures of *Amphora* sp. and *Porphyridium* sp. settlement on the glass slides and the AF coatings embedded with the CT-6/TJ-2; adhesive density of (**B**) *Amphora* sp. and (**C**) *Porphyridium* sp. TJ-2 was extracted from Z. *armatum* DC fruit. (*p* values: 0.01 < * *p* < 0.05, *** *p* < 0.001) Reprinted with permission from [45].

**Figure 17:**
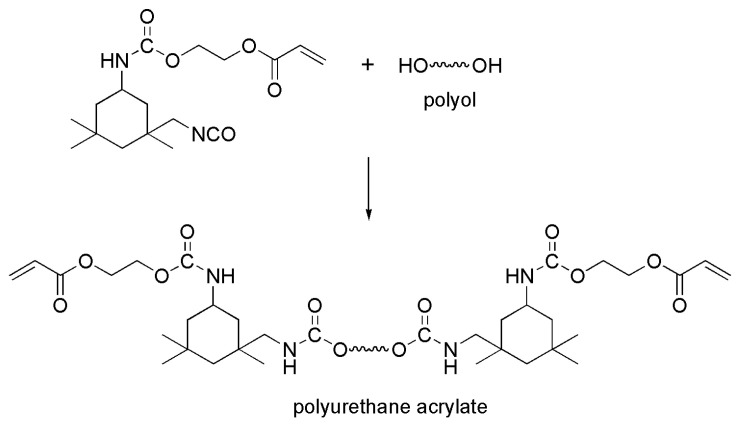
Preparation of PUA resin. Reprinted with permission from [64].

Moreover, the use of natural products may have unknown effects on the marine environment [11]. In addition to evaluating the AF properties of natural products, their biological activities and biocompatibilities need to be assessed to evaluate whether they can be used as potential AF candidates. Vanessa Agostini’s group studied the AF properties of twelve aqueous plant extracts from the Brazilian semiarid biome with different concentrations, and investigated whether extracts are safe to nontarget organisms [63]. The result showed that these extracts of *Harpochilus neesianus* mix, *Myracrodruon urundeuva* leaves, *Byrsonima gardneriana* leaves, *Sideroxylon obtusifolium* branches and *Turnaera hermannioides* leaves reduced the biofilm bacterial density (≥80%) and biofilm biomass. In addition, it was found that *Harpochilus neesianus* mix is nontoxic to plantonic bacterial, and *Turnaera hermannioides* is not toxic to the microalgae and crustacean species. These extracts are promising alternatives to traditional AF paints in the future. Zhou’s group extracted and isolated a series of active components from *Zanthoxylum bungeanum* (*Z. bungeanum*) and demonstrated their antibiofouling characteristic and nontoxic to organisms through antibarnacle larvae experiments and antialgae tests. Subsequently, this group fabricated the self-polishing resin by free radical polymerization, in which the functionalized triclosan with antialgae effect was copolymerized with general acrylic molecules. The essential antiadhesion tests and quartz crystal microbalance (QCM-D) adsorption experiments proved that the CT-6 isolated from *Z. bungeanum* leaves had nontoxic and broad-spectrum antiadhesion properties (effectively inhibited the adhesion of *Amphora* sp. and *Porphyridium* sp.) (Figure 16) [45]. Sheshtawy’s group synthesized the polyurethane acrylate (PUA) polymer through the addition reaction between an isophorone diisocyanate (IPDI) and 2-hydroxyethyl acrylate (Figure 17) [64]. Subsequently, they prepared the polyurethane acrylate (PUA)/natural filler-based composite (rhizome water extract of *Costus speciosus*) as an AF agent. The *E. coli and P. aeruginosa* cell growth assays showed that well-dispersion of natural fillers in the PUA polymer (2 wt%) potently reduced the number of microorganism strains, indicating that the PUA/natural filler composite might be considered an ecofriendly and economical solution to the biofouling problem. These works are helpful for the development of nontarget biosafety AF coatings in the future.

Based on these latest research results, it can be seen that the AF properties of the compounds are affected by molecular structures. For example, isothiocyanates with different enantiomers (such as isocyano groups, acetamide groups, amide groups and halogen) exhibited different AF activities [46]. The number of hydrophobic biphenyl groups and hydrophilic cationic arginine sidechains affected the lipophilicity of DKPs. The higher the lipophilicity, the compounds tend to bind to the cell membrane of fouling organisms to achieve an antifouling effect. The lower the lipophilicity, the more hydrophilic the compound exhibits, and the weaker the interaction with fouling organisms [37]. The AF performance of extracts isolated from the soft coral *Sinularia flexibilis* was influenced by the hydroxyl and double bonds. Hydroxyl group decreased the AF activity for *Bugula neritina* [35]. Targett compared the antialgae activity of picolinic acid, nicotinic acid and pyridine, and found that carboxyl groups had a significant influence on the AF performance rather than N-methylation of the pyridine nucleus [65]. It shows that the type and location of functional groups have different effects on the AF properties of natural agents, and the influence mechanism is more complex.

## 4. Conclusions

In summary, we have summarized the recent research progress of natural product extracts from marine organisms and terrestrial plants as AF agents in the past five years. Key structural units of the extracts from marine organisms mainly include terpenoids, diterpenoids, phenolic compounds, monoterpene–furan hybrid molecules, pyrone-type compounds, peracetylated cholic acid, alkaloids, diketopiperazine and their derivatives. Key structural units of the extracts from terrestrial plants mainly include isothiocyanate compounds, natural organic acids, tannic acid, polylactic acid, capsaicin and their derivatives. These natural extracts as AF agents and some novel strategies for preparing protective coatings with high AF activity and nontoxicity have been mentioned as guidance for developing a new generation of AF agents. With the rapid development of science and technology, it is believed that we will have a clearer understanding of the mechanism of natural AF agents in the near future and have a more optimized process for controlling the release or leaching speed of AF agents. Natural products can be better applied to AF coatings.

## Figures and Tables

**Table 1:** Name, resources and targets of some compounds from algae.

No.	Compounds’ Name	Resources	Targets	Ref.
1	(+)-Catechin	*Posidonia oceanica*	*Phaeodactylum tricornutum* *Aliivibrio fischeri* *Navicula salinicola * *Ficopomatus enigmaticus*	[17]
2	Ferulic acid			
3	Epicatechin			
4	Chlorogenic acid			
5	Gallic acid			
6	Aplysin-20 aldehyde	*Laurencia venusta*	*Mytilus galloprovincialis*	[22]
7	13-dehydroxyisoaplysin-20			

**Table 2:** Name, resources and targets of some compounds from marine invertebrates.

No.	Compounds’ Name	Resources	Targets	Ref.
1	9,11-dihydrogracilin A	*Dendrilla antarctica*	*Botrylloides* sp.	[28]
2	9,11-dihydrogracillinone A			
3	Peracetylated cholic acid	*Siphonochalina fortis*	*Mytilus edulis platensis*	[29]
4	2,5-diketopiperazine	*Geodia barretti*	-	[30]
5	dihydrofurospongin II (2)	Mediterranean sponges	*Balanus (Amphibalanus) amphitrite*	[31]
6	euryfuran			
7	Phidianidine A	*Phidiana militaris*	-	[32]
8	Sarcoglaucin B	*Sarcophyton glaucum*	*Balanus amphitrite*	[33]
9	sarcoglaucin E			
10	trochelioid			
11	7α-hydroxy-Δ^8(19)^-deepoxysarcophine			
12	(−)-sartrochine			
13	H12-Vibrio alginolyticus	*Pocillopora damicornis*	*Pseudomonas aeruginosa*	[34]
14	ent-sinuflexibilin D	*Sinularia flexibilis*	*Bugula neritina*	[35]
15	sinulaflexiolide O			
16	sinulaflexiolide L			
17	diepoxycembrene A			
18	orphirin and sinensin			

**Table 3:** Name, resources and targets of some compounds from terrestrial plants.

No.	Compounds’ Name	Resources	Targets	Ref.
1	*Stellera chamaejasme* extracts	*Stellera chamaejasme*	*Porphyridium* sp.	[20]
2	N-methyltetrahydroellipticine	*Aspidosperma austral*	*Mytilus edulis platensis*	[44]
3	furoquinoline alkaloids kokusaginine	*Balfourodendron riedelianum*		
4	flindersiamine			
5	CT-6(extracted from leaves)	*Zanthoxylum bungeanum*	*Navicula* sp. *Amphora* sp. *Porphyridium* sp.	[45]
6	TJ-2(extracted from fruit)			
